# The efficacy of aspirin and metformin combination therapy in patients with rectal aberrant crypt foci: a double-blinded randomized controlled trial

**DOI:** 10.1186/s12885-020-07564-z

**Published:** 2020-10-29

**Authors:** Takuma Higurashi, Jun Arimoto, Keiichi Ashikari, Tomohiro Takatsu, Noboru Misawa, Tsutomu Yoshihara, Tetsuya Matsuura, Akiko Fuyuki, Hidenori Ohkubo, Atsushi Nakajima

**Affiliations:** Department of Gastroenterology and Hepatology, Yokohama City University School of Medicine, 3-9 Fukuura, Kanazawa-ku, Yokohama, 236-0004 Japan

**Keywords:** Chemoprevention, Aspirin, Metformin, Combination therapy, Aberrant crypt foci

## Abstract

**Background:**

The incidence and mortality rates of colorectal cancer (CRC) continue to increase worldwide. Therefore, new preventive strategies are needed to lower the burden of this disease. Previous studies reported that aspirin could suppress the development of sporadic colorectal adenoma. In addition, metformin is a biguanide derivative that is long widely used for the treatment of diabetes mellitus and has recently been suggested to have a suppressive effect on carcinogenesis and cancer cell growth. Both drugs exhibit a chemopreventive effect, but their efficacy is limited.

Aberrant crypt foci (ACF), defined as lesions containing crypts that are larger in diameter and stain more darkly with methylene blue than normal crypts, are more prevalent in patients with cancer and adenomas, and considered a reliable surrogate biomarker of CRC. Thus, we designed a prospective trial as a preliminary study prior to a CRC chemoprevention trial to evaluate the chemopreventive effect of aspirin combined with metformin on colorectal ACF formation in patients scheduled for polypectomy.

**Methods:**

This study is a double-blind randomized controlled trial that will be conducted in patients with both colorectal ACF and colorectal polyps scheduled for polypectomy. Eligible patients will be recruited for the study and the number of ACF in the rectum will be counted at the baseline colonoscopy. Then, the participants will be allocated to one of the following two groups; the aspirin plus placebo group or the aspirin plus metformin group. Patients in the aspirin plus placebo group will receive oral aspirin (100 mg) and placebo for 8 weeks, and those in the aspirin plus metformin group will receive oral aspirin (100 mg) and metformin (250 mg) for 8 weeks. After 8 weeks of administration, polypectomy will be performed to evaluate changes in the number of ACF, and the cell-proliferative activity in the normal colorectal mucosa and colorectal polyps.

**Discussion:**

This is the first study proposed that will explore the effect of aspirin combined with metformin on the formation of colorectal ACF in humans.

**Trial registration:**

This trial has been registered in the University Hospital Medical Information Network (UMIN) Clinical Trials Registry as UMIN000028259. Registered 17 July 2017.

## Background

Cancer is a major health concern and the leading cause of death worldwide. The incidence and mortality of colorectal cancer (CRC) continues to increase worldwide [1]. Most cases of CRC originate from adenomas [2], and their removal has been shown to reduce the risk of future development of CRC and advanced adenoma [3, 4], thereby preventing CRC-related death [5]. However, patients with adenomas and CRC constitute a high-risk group for the development of metachronous colorectal adenoma and/or CRC [6]. Therefore, a paradigm shift from surveillance for the early detection of cancer or adenomas and polypectomy to new tactics for prevention, including chemoprevention, is required to lower the burden of this disease. Several large epidemiologic and clinical studies have evaluated the possible effects of more than 200 agents, including fiber, calcium, and non-steroidal anti-inflammatory drugs (NSAIDs), such as aspirin and selective cyclooxygenase-2 (COX-2) inhibitors, in protecting against CRC development [7]. Previous studies reported that aspirin suppressed the development of sporadic colorectal adenoma [8–10]. However, the chemopreventive effect of aspirin is limited, and these studies also reported that aspirin increased gastrointestinal bleeding. In these patients, a post-aspirin chemoprevention drug is then needed to establish CRC chemoprevention.

Patients with type 2 diabetes who were prescribed metformin have been reported to be at a low risk of cancer development, including CRC), compared with those who were not treated with metformin [11, 12]. This evidence suggests that metformin might be a candidate agent for CRC chemoprevention in diabetic patients. In previous studies, we demonstrated the chemopreventive effect of metformin in two rodent models (a genetic model and a chemically-induced cancer model) and two human studies of colorectal carcinogenesis. We demonstrated that metformin inhibited the development of intestinal polyps in adenomatous polyposis coli mice, a murine model of familial adenomatous polyposis [13]; furthermore, we demonstrated that metformin inhibited azoxymethane-induced formation of colorectal aberrant crypt foci (ACFs) by activating AMP-activated protein kinase (AMPK) [14]. Both studies were conducted in nondiabetic mice, which suggested the direct chemopreventive potential of metformin per se. We also conducted a trial involving non-diabetic human patients and showed that oral low-dose metformin (250 mg/day) was safe and suppressed the formation of colorectal ACF. In the study conducted on nondiabetic human subjects, the drug was safe [15]. Based on these findings, we performed a randomized clinical trial (RCT) and showed that low dose metformin is safe and reduced the incidence of new polyps in patients after polypectomy of the colon [16]. In that trial, we showed the safety and chemopreventive effect of metformin on colorectal carcinogenesis. However, similar to aspirin, the chemopreventive effective was limited.

The current recommended treatment for hypertension or diabetes mellitus is multiple drug combination therapy, which requires a lower dose of each drug to achieve a maximum therapeutic effect while avoiding toxic effects. Here, we hypothesize that aspirin and metformin combination therapy is more effective than single use of these drugs for CRC prevention. To test this hypothesis, this proposed study will investigate whether the combined use of aspirin and metformin shows a stronger chemopreventive effect than aspirin or metformin alone. In CRC chemoprevention trials, in general, the incidence of adenomas or the cancer itself is set as the study endpoint. Although the incidence rate of CRC is the most reliable endpoint, the use of this endpoint would be unsuitable for chemoprevention trials because of the relatively low occurrence rate of CRC in the general population [17] and the long-term observation period that it would require. ACF are defined as lesions containing crypts that are larger in diameter and stain more darkly with methylene blue than normal crypts [18–21]. They have been reported to be more prevalent in patients with cancer and adenomas, are reduced by the use of chemopreventive drugs such as NSAIDs, are considered a reliable surrogate biomarker of CRC [22, 23]. We previously reported the usefulness of ACF as a biological marker of CRC [24, 25], and conducted a chemoprevention trial for colorectal ACF [15, 26]. The advantages of chemoprevention trials using colorectal ACF as the primary endpoint are that long-term observation is not required to evaluate the drug effect, and ACF can be estimated quantitatively. Thus, we set ACF as a suitable endpoint for this trial. To the best of our knowledge, this is the first clinical trial to investigate aspirin and metformin combination therapy as a chemopreventive strategy in patients with colorectal ACF.

## Methods/design

### Study design and setting

This study is designed as a double-blind placebo-control RCT to be performed in nondiabetic patients with both colorectal ACF and resectable polyps. It will be conducted in the Department of Gastroenterology and Hepatology at Yokohama City University (YCU) Hospital. The coordinating office will be at the YCU Hospital, and registration and data collection will be conducted at the YCU center for novel and exploratory clinical trials (Y-NEXT).

### Ethical considerations and registration

The study protocol complies with the Declaration of Helsinki [27] and the Ethics Guidelines for Clinical Research published by the Ministry of Health, Labor, and Welfare, Japan [28]. Patients and the public were not involved in the study design. We obtained approval for this study from the Ethics Committee of Yokohama City University Hospital on December 22, 2016. The protocol and informed consent form were approved by the institutional ethics committee at Yokohama City University Hospital. This trial has been registered in the University Hospital Medical Information Network (UMIN) Clinical Trials Registry as UMIN000028259. Written informed consent for participation in the study will be obtained from all participating patients. The trial results will be reported in conformity with the Consolidated Standards of Reporting Trials (CONSORT) 2010 guidelines [29].

### Eligibility criteria

Patients with both colorectal ACF and resectable polyps will be recruited for this study. The inclusion criteria are as follows:

1. Patients with resectable polyps.
2. Patients with more than 10 rectal ACF.
3. Willingness to provide written informed consent.

The exclusion criteria are as follows:

1. Patients with lesions for which preferred early resection is preferred.
2. History of regular use (defined as at least once per week) of NSAIDs and/or aspirin.
3. History of regular use of warfarin and/or direct oral anticoagulants (DOAC).
4. History of diabetes mellitus (defined as a glycosylated hemoglobin (HbA1c) level more than 6.5% or regular use of anti-diabetic drugs).
5. History of heart failure, renal failure, liver cirrhosis or chronic hepatic failure.
6. History of familial adenomatous polyposis, hereditary non-polyposis CRC and inflammatory bowel disease.
7. Pregnancy or possibility of pregnancy.
8. Contraindication to aspirin or metformin.
9. Allergy to aspirin or metformin
10. Patients judged as inappropriate candidates for the trial by the investigators.

### Intervention

All eligible patients will be randomly allocated to one of the two following groups; the aspirin plus placebo group and aspirin plus metformin group. Endoscopists, doctors at the follow-up outpatient clinics and patients will be blinded to the groups. Patients in the aspirin plus placebo group will receive oral aspirin (100 mg) and placebo per day for 8 weeks, and patients in the aspirin plus metformin group will receive 100 mg of aspirin and 250 mg of metformin per day for 8 weeks. At the end of the 8 weeks of administration of a colonoscopy will be performed to evaluate the number of rectal ACF.

### Outcome measurements

The primary endpoint will be the change in the number of colorectal ACF after 8 weeks of treatment. A magnifying colonoscope will be used in all cases (H260AZI, PCF-Q260AZI, PCF-Q290AZI, HZ290; Olympus Co., Tokyo, Japan), with carbon dioxide insufflation. Bowel preparation for the colonoscopy will be initiated one day before the procedure. Each patient will be instructed to consume a low-residue diet and take 5 mg of oral sodium picosulfate on the evening before the procedure. On the day of the procedure, each patient will be given 1500 ml of polyethylene glycol (PEG). If the stools are not sufficiently clear, an additional 500 ml of PEG will be given to ensure sufficient bowel cleaning. For conscious sedation, midazolam and pentazocine will be administered at the beginning of the procedure. Intramuscular glucagon or scopolamine will be administered to reduce colonic movements. At the time of the first colonoscopy, the endoscope will be inserted into the cecum, and the entire colorectum will be carefully observed as the endoscope is pulled back. Rectal epithelial samples will be obtained. The number of rectal ACFs will be counted as follows. First, the rectum will be washed with warm water, then sprayed with 0.25% methylene blue solution and stained for 2 min, washed again with warm water, and finally examined by magnifying endoscopy for ACFs. At the end of 8 weeks of administration, the same endoscopists will perform the polypectomy and counting of the ACF. All procedures will be recorded on DVD, and all of the ACF will be photographed. The number of ACF in each patient will be counted by the endoscopists during the colonoscopy. To further ensure validity, the number of ACF will be counted again through observation of the recorded DVD by 3 blinded expert endoscopists (H.T, A. J and A.K). If there was a discrepancy among the blinded specialists, we adopted the consent of at least two of the three. Additionaly if these expert endoscopists judge the colonoscopy examination as inadequate, that case will be excluded.

The secondary outcomes will include (1) the drug safety; adverse events (AEs) will be graded according to the National Cancer Institute Common Toxicity Criteria for Adverse Events (NCI-CTCAE), version 4.0. All study participants will be provided with a study diary to record the daily dosage of the study treatment and the AEs. Patients developing grade 3 or more severe AEs will be withdrawn from the study at that point. (2) Effects of additional metformin on the cell-proliferation in the rectal epithelium and polyps: Rectal epithelial samples will be obtained from the same trial patients by biopsy at the time of the first colonoscopy and polypectomy. Cell-proliferative activity will be evaluated by the analysis of the Ki-67 labeling indices. Briefly, immunochromatography will be carried out using a50-fold dilution of the Ki-67 antibody (DAKO, Glostrup, Denmark) and the avidin-biotin-peroxidase complex (ABC) kit (Vector, Burlingame, CA) according to the manufacturer’s instructions. Then, we will randomly select six crypts and count the number of Ki67-positive cells per crypt. In total, ~ 250 cells will be counted at a magnification of × 400 using a bright-field microscope. The results will be presented as the percentage of Ki67-positive cells. All participants will undergo a physical examination and laboratory tests at the time of the baseline endoscopic examination and polypectomy.

### Randomization

The investigator will repot the patient’s details to the central registration center via fax. After an eligibility check, the patients will be randomly assigned to receive aspirin plus placebo or aspirin plus metformin at the central registration center by a computer program that will block allocation by age and sex. In this way, the patient assignments will be concealed from the investigator. The randomization center will allocate a numbered treatment pack to each patient, which will contain all the drugs or placebos needed to complete a course of the trial treatment for one patient. Drug allocation was masked from all patients, endoscopists, doctors, and investigators until the end of the trial.

### Drug supply

Aspirin will be purchased from Bayer Pharma, Ltd. Metformin will be purchased from Dainippon Sumitomo Pharma Co., Ltd. The placebo (250 mg lactose) will be purchased from Kondo photo process Co., Ltd., Osaka, Japan. All trial drugs will be packaged identically and identified only by number. Subjects will be instructed to take two tablets of the trial drugs after breakfast each day. Compliance will be monitored by counting the empty drug packages returned by the patients at colonoscopy. The participants will be also interviewed and monitored that they had not used prohibited agents (aspirin, metformin and/or other non-steroidal anti-inflammatory drugs). If serious adverse events or less than 80% drug compliance are confirmed in a patient, that patient will be removed from the final analysis.

### Sample size estimation

We previously showed that metformin administered at 250 mg/d for 1 year suppressed metachronous adenoma and the effectiveness was similar to previous aspirin chemoprevention trials [8, 9, 16]. Based on these finding, we estimated that aspirin and metformin have similar chemopreventive effects on colorectal carcinogenesis. Then we estimated that the ACF number would change about − 3 ± 2 (mean ± SD) in the aspirin plus placebo group and − 5 ± 2 (mean ± SD) in the aspirin plus metformin group based on our previous metformin ACF prevention trial [15]. To detect the reduction in the number of ACFs in the two groups using the Student’s *t* test with a two-sided significant level of 5% and a power of 80%, it was found that a sample size of 17 to 26 patients in each group would be necessary. Assuming some patients would dropout, we propose to recruit a total of 60 patients with 30 patients in each group.

### Statistical analysis

The changes of ACFs number in each group, the primary endpoint, will be compared between the aspirin plus placebo group and the aspirin plus metformin group by the Student *t* test. The safety, one of the secondary endpoints, will be compared by the chi-square test. The remaining results in the two groups will be compared by the Mann-Whitney *U* test or the *t*-test. A *P* values of < 0.05 will be regarded as indicative of statistical significance. The analysis will be performed using SPSS statistics, version 26 (SPSS, Chicago, IL, USA).

### Trial steering committee and data monitoring committee

The Trial Steering Committee and Data Monitoring Committee will be located in the Department of Gastroenterology and Hepatology at Yokohama City University Hospital. The Management Team will monitor the trial progress status and data by face-to-face and/or telephonic contact with each of the trial investigators every month.

### Study flow

**Fig. 1:**
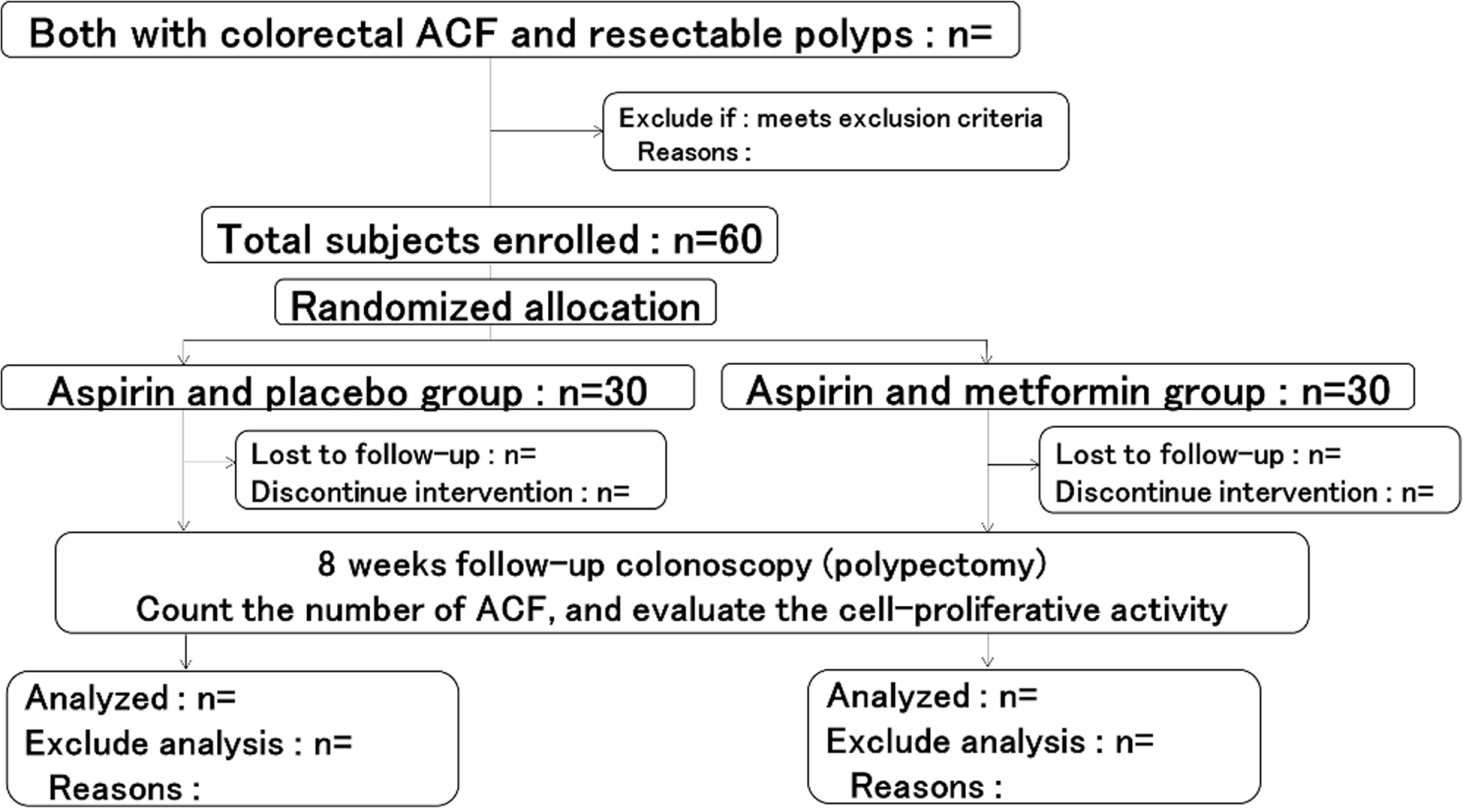
Study Flow

A flow chart of the study is shown in Fig. 1.

## Discussion

This is the first study proposed to evaluate the chemopreventive effect of aspirin and metformin combination therapy in patients with rectal ACF. Given its role as an analgesic, antipyretic and agent for cardiovascular prophylaxis, aspirin has become one of the most commonly used drugs. Many studies have provided considerable evidence demonstrating its potential for the prevention of CRC. Aspirin exerts its anticancer effects through several interconnected mechanisms, including prostaglandin synthesis and catabolism in epithelial cells [30, 31]; inhibition of WNT β-catenin signalling [32, 33]; inactivation of platelets [34, 35] and the host immune response [31, 36]. Aspirin may also act through several other unknown mechanisms. Furthermore, aspirin may has been hypothesized to act synergistically with other agents [37]. Further study is needed to clear the chemopreventive effect of aspirin. Metformin (1,1-dimethylbiguanide hydrochloride) is a biguanide derivative that is widely used for the treatment of diabetes mellitus [38]. Metformin activates AMPK, which inhibits the mammalian target of rapamycin (mTOR) pathway [39]. The mTOR pathway plays an important role in the regulation of cellular protein translational machinery and cell proliferation [40]. The best-characterized downstream effector of mTOR is S6 kinase, which regulates the initiation and elongation phases of translation [41]. Activation of the mTOR pathway has been shown to accelerate cell cycle progression from G1 to S in CRC DLD-1 cells [42]. Therefore, AMPK activation may inhibit cell growth and proliferation by suppressing protein synthesis, thereby having a potent antiproliferative effect. Recent evidence indicates that metformin has a suppressive effect on tumorigenesis and cancer cell growth [43–45]. In one study, metformin was demonstrated to activate AMPK and consequently decrease cellular proliferative activity, to produce a general decrease in protein synthesis in vitro in human breast carcinoma cells [43]. Metformin was also shown to inhibit the proliferation of human prostate cancer cells [45]. In addition, we performed a RCT and we showed that metformin reduced the incidence of new polyps in patients after polypectomy of the colon [16]. However, similar to aspirin, the chemopreventive effective was limited. In this study, we aim to evaluate the chemopreventive effect of aspirin and metformin combination therapy in patients with rectal ACF and determine whether the combined use of aspirin and metformin produces a stronger chemopreventive effect than each drug alone. Because we hypothesize that both aspirin and combination therapy may have a chemopreventive effect, a higher number of baseline ACFs (> 10) was needed to detect a difference between the two treatment effects. We previously reported a mean number of ACFs in adenomas of 6.2 ± 7.0 [24], which is why we set > 10 ACFs as an inclusion criterion. Consequently, we expect the trial to take longer because of the small number of patients who meet the inclusion criteria.

This trial may have the following limitations. First, ACFs are considered as a reliable surrogate biomarker of CRC [22], although their biological significance still remains controversial. However, setting study endpoint as ACF has large merit to reduce efforts because ACFs are quantitative and it is possible to observe changes in a short period of time. Thus, we devised a trial using ACF as the primary endpoint to evaluate the chemopreventive effects of aspirin and metformin combination therapy. Second, an intervention period of 8 weeks may be too short to allow the reliable detection of differences between the groups. However, we showed in a previous study that oral administration of metformin for 1 month suppressed the formation of colorectal ACF in humans. Other reports show that ACFs decrease in number in 8 weeks with the use of NSAIDs [23]. Therefore, we think that an intervention period of 8 weeks would be sufficient to evaluate the changes in the number of ACF. We previously conducted a short-term chemoprevention trial of metformin for colorectal ACF, and we showed the suppressive effect of the drug on the formation of ACF. Thereafter, we conducted a long-term metformin chemoprevention trial for colorectal polyps. We propose to repeat the same steps for the chemoprevention trial to investigate the combined use of aspirin and metformin. Third, our study lacks dose-response data. However, low-dose aspirin (100 mg/d) is used worldwide with reported chemopreventive effects on CRC and adenoma. Furthermore, we previously reported the effect of low-dose metformin (250 mg/d) on colorectal adenoma and ACF. Finally, our study lacks a metformin alone arm and double placebo arm, while the use of aspirin alone has not been established to suppress to the formation of ACFs. However, aspirin is one of the most efficiacious chemopreventive agents for the treatment of colorectal adenoma and CRC, which is why we used aspirin alone as the control arm in the present study. A four-arm study using a double placebo is needed in the future to investigate the combination effect of metformin and aspirin.

If this combination therapy was found to be more effective for the prevention of CRC, the impact would be significant. Therefore, we consider it of interest to determine whether the combined use of aspirin and metformin shows a stronger chemopreventive effect on the formation of human colorectal ACFs than either aspirin or metformin alone.

## Data Availability

The datasets used and/or analyzed during the current study will be available from the corresponding author on reasonable request.

## Abbreviations

CRC: Colorectal cancer
NSAIDs: non-steroidal anti-inflammatory drugs
COX-2: cyclooxygenase-2
ACF: Aberrant crypt foci
AMPK: AMP-activated protein kinase
UMIN: University hospital Medical Information Network
CONSORT: Consolidated Standards of Reporting Trials
